# Supplementary material for: Development of an Optimized MALDI-TOF-MS Method for High-Throughput Identification of High-Molecular-Weight Glutenin Subunits in Wheat
Source: Molecules. 2020 Sep 22;25(18):4347. doi: 10.3390/molecules25184347 (PMC7571055; doi:10.3390/molecules25184347)
Supplement: Supplementary file 1 [file molecules-25-04347-s001.zip › molecules-935056 Supplementary Figure.pdf]

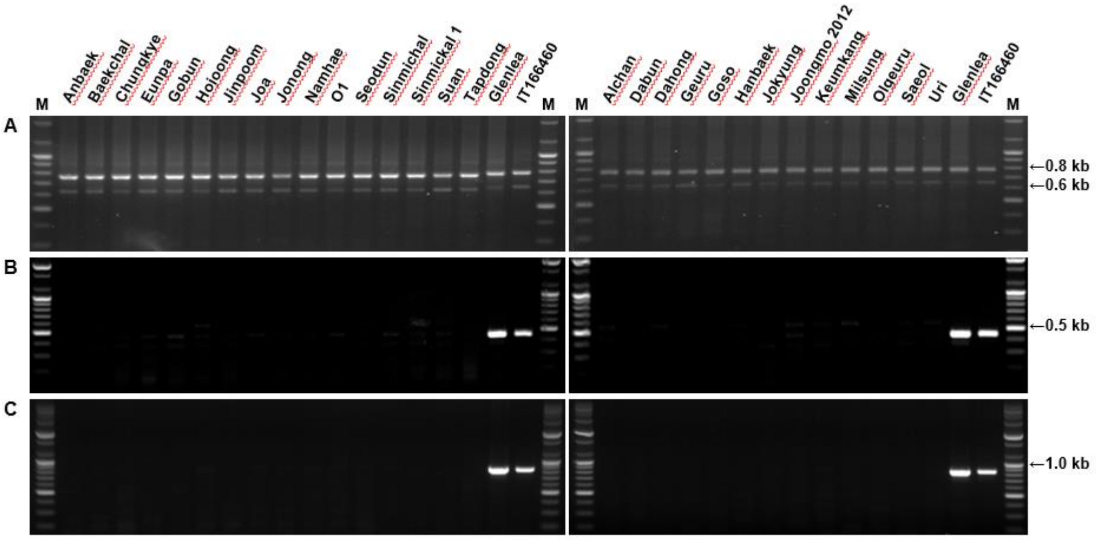

Figure S1. Amplification of DNA from Korean cultivars containing 1Bx7 group 1 or 1 Bx7 group 2 HMW-GS and standard cultivars containing 1Bx7OE. (**A**) PCR primers span the region of the 18 bp insertion in 1Bx7OE; (**B**) PCR primers from the left junction of the retroelement and duplicated region of Bx7OE; (**C**) PCR primers from right junction of the retroelement and duplicated region of Bx7OE. Glenlea and IT166460 were used as positive controls for Bx7OE. The 100bp Plus DNA Ladder is shown in lanes (**M**).
